# Supplementary material for: Action and Entropy in Heat Engines: An Action Revision of the Carnot Cycle
Source: Entropy (Basel). 2021 Jul 5;23(7):860. doi: 10.3390/e23070860 (PMC8304742; doi:10.3390/e23070860)
Supplement: Supplementary file 1 [file entropy-23-00860-s001.zip › supplementary-1.pdf]

Addendum: Justification from statistical mechanics that entropy and Gibbs energy is an exact function of action.

Boltzmann's statistical definition of entropy defined the probability of finding a particular arrangement of particles in different energy levels as the quotient of the total number of possible permutations divided by the product of the number of permutations in each level. This result was the basis of statistical mechanics as developed later by Gibbs, allowing Boltzmann to relate statistical entropy to the thermal entropy defined earlier by Clausius ($S = \int dQ/T$).

The following analysis uses notation given by Walter Moore (1962), taking care to distinguish between the partition function for the Gibbs *canonical* ensemble Z equivalent to the summation $\Sigma g_j e^{-Ej/kT}$ involving the internal energy E per mole and the partition function z (lower case) symbolising the *microcanonical* ensemble equivalent to the singular state summation $\Sigma g_j e^{-\varepsilon j/kT}$.

Boltzmann's famous conjecture regarding the relation of entropy to diversity in arranging the energy of N particles is given in (i).

$$S = k\ln W = \int dQ/T \qquad \text{(i)}$$

where

$$W = N!/(N_1!\, N_2!\, N_3!\, N_4! \,....N_j!) \qquad \text{(ii)}$$

Using Stirling's approximation for the logarithm of a factorial, we have (where ω is a normalizing factor),

$$\ln W = (N_1 \ln N_1 + N_2 \ln N_2 + N_3 \ln N_3 \,...)\omega + C \qquad \text{(iii)}$$

According to Boltzmann (1896), "the most probable distribution of velocities of the molecules in our cells will be the one for which ln$W$ is a maximum". At this equilibrium point the colliding particles are considered (Boltzmann, 1896) to scatter into new energy cells while conserving energy. Cells differing most in energy are the least probable. Without collisions establishing equilibrium for the Maxwell-Boltzmann distribution, minimizing the difference between neighbouring levels, maximum entropy as expressed in $k\ln W$ would be impossible. Boltzmann also equates Clausius' version of entropy as the reversible addition of heat with the following formula involving an exponential of temperature ($T^{3/2}$) and the inverse of partial density (ρ^{-1}), the equivalent of specific volume.

$$S = \int dQ/T = k\ln[T^{3/2}\rho^{-1}] + \text{constant} \qquad \text{(iv)}$$

In his *H*-theorem and in his discussion of how it is related to irreversibility and increasing entropy and initial conditions, Boltzmann describes the evolutionary behavior of a function of the form H equal to the integral $\int f \ln f \, d\omega$, where f is a function taking on the role of relative probability.

From Boltzmann's relationship, using Stirling's approximation for the logarithm of a factorial we have entropy as follows (v).

$$S = k(N\ln N - N) - k\Sigma(N_j \ln N_j - N_j) \qquad \text{(v)}$$

Influenced by Clausius and Boltzmann, Gibbs defined the microcanonical ensemble for a system (i.e. molecule) of fixed energy (ε_j), volume (V) and particle number (N). Substituting in equation (v) for Nj = $Ng_je^{-\varepsilon j/kT}/\Sigma g_je^{-\varepsilon j/kT}$ and taking $\Sigma g_je^{-\varepsilon j/kT}$ as the microcanonical partition function z, where g_j is the occupancy or degeneracy of level j, we obtain,

$$S = k(\mathrm{NlnN–N}) – k\mathrm{N}(\ln\mathrm{N} – \ln z – 1 – \varepsilon_j/kT) \quad \text{(vi)}$$

$$S = \mathrm{N}\varepsilon_j/T + k\mathrm{N}\ln z \;=\; \mathrm{E} + \; k\ln z^{\mathrm{N}} \quad \text{(vii)}$$

The molecular partition function z can be considered as representative of a system's trajectory independent of time, since its energy state is fixed. The relationship between the canonical and microcanonical partition functions is established by the following simplified relationship (viii), indicating different ways of assigning particles to the energy levels, determining the distinct state of the system.

$$Z = \Sigma e^{-Ej/k\,T} \;=\; \Sigma e^{(-\varepsilon j(1) + \varepsilon j(2) + \varepsilon j(3) \ldots\ldots \varepsilon j(N))/kT)} \;=\; \Sigma e^{(-\varepsilon j(1)/kT} e^{-\varepsilon j(2)/kT} e^{-\varepsilon j(3)/kT} \ldots\ldots e^{-\varepsilon j(N))/kT)} \quad \text{(viii)}$$

Since each particle has the same set of allowed energy levels this sum is equal to (ix).

$$Z \;=\; (\Sigma_j e^{-\varepsilon j/kT})^N \;=\; z^N \quad \text{(ix)}$$

For example, consider a simple case of two particles (1) and (2) and two energy levels ε_1 and $\varepsilon_{2.}$ These can be assigned in four ways. The sum over states is given by (x).

$$Z = e^{-E1/kT} + \; e^{-E2/kT} + e^{-E3/kT} + \; e^{-E4/kT} \quad \text{(x)}$$

which is equal to (xi).

$$e^{-2\varepsilon 1/kT} \;+\; 2e^{-\varepsilon 1/kT}e^{-\varepsilon 2/kT} + \; e^{-2\varepsilon 2/kT} \;=\; (\Sigma e^{-\varepsilon j/kT})^2 = z^N \quad \text{(xi)}$$

Gibbs' canonical ensemble of systems free to fluctuate in energy content with time, had a partition function for many systems in the same heat bath; here, where particles of gas or liquid are free to explore the entire volume, a divisor of N! must be included to correct the number of distinguishable states (Moore, 1962), given the particles are identical.

$$Z = z^N/\mathrm{N}! \quad \text{(xii)}$$

This is an important distinction regarding independence of molecular systems applying only to ideal gases free of binding interactions. To the extent a system is liquid or solid, this distinction no longer applies since the individual molecules can be distinguished by location. Furthermore, for ideal or dilute gases the vast majority of energy levels j at ambient temperatures or higher will have only single occupancy (i.e. unit degeneracy with $g_j = 1$), we can take $\Sigma e^{-\varepsilon j/kT} = z$.

From quantum mechanics for a particle in a box of volume V, we have translational energy levels with quantum number n, a cubic partition function z is determined (Moore, 1962) as follows.

$$z \;= \Sigma e^{-(n2h2/8ml2)/kT} = V(2\pi mkT)^{3/2}/h^3 \quad \text{(xiii)}$$

$= \mathrm{N}a^3\{2\pi m^2\ddot{u}^2/3\}^{3/2}/h^3$ where $V = \mathrm{N}a^3$ and $\ddot{u}$ is Maxwell's root-mean-square velocity

$= \mathrm{N}r^3\{(\pi mv)^2\}^{3/2}/h^3$ where $a = 2r$ and $\ddot{u}^2 = 3\pi v^2/8$ where v is the mean speed

$= \mathrm{N}(mrv/2\hbar)^3 = \mathrm{N}n_t^3$ $\qquad \hbar = h/2\pi$ (xiv)

Replacing the root-mean-square velocity ü with the mean speed v in a Maxwell-Boltzmann distribution (Brown, 1968) and inserting a symmetry factor of 2 to prevent double counting of the particles action eliminated the need for a previous z_t correction factor ($10.22967=1.0854^3 x 2^3$, Kennedy at al., 2015, 2019) in the denominator. For the calculations in Tables 1, 2 and 3, n_t^3 is of the order of 10^5 to 10^6, indicating the high nondegeneracy and the freedom of action of the molecules. They are far from confined to the minimal action volume defined by $\hbar^{3.}$ According to Boltzmann, these Carnot cycle systems are highly disordered, compared to having an n_t^3 nearer 1 when phase space is minimal. Then we can interpret the microcanonical translational partition function $\Sigma e^{-\varepsilon j/kT}$ as the total cubic action space, as long as the temperature is sufficiently high.

$$z \quad = Nn_t^3 \qquad \text{(xv)}$$

So, noting this result for the molecular partition reflects an inverse of the scale of the degeneracy for each energy level, the levels are easily accessed for translation by collisions, given the translational energy barrier for transition from one level to another is easily exceeded by kT. Indeed, at equilibrium only the levels very near to the average energy are occupied (Schrödinger, 1952). Under these environmental conditions of temperature and pressure we then have the partition function as reflecting the cubic action, validating the classical procedure used to calculate entropy from action used in this paper.

Then for the canonical partition function for multiple systems we find,

$$Z \quad = \Sigma_j e^{-Ej/kT} = z^N/N! \; = \; (Nn_t^3)^N/N! \qquad \text{(xv)}$$

Using Stirling's approximation that $N! \equiv (N/e)^N$,

$$Z \quad = (n_t^3 eN)^N/N^N = (n_t^3 e)^N$$

Taking the logarithm preceding with $-kT,$ we can obtain the Helmholtz energy (A)

$$-kT\ln Z = A$$

$$= -kT\ln(n_t^3 e)^N$$

$$= -NkT\ln[(n_t)^3] - NkT \qquad = G \; - RT \qquad \text{(xvi)}$$

Schrödinger (1952) remarked that $-NkT\ln(1/\zeta)$, where zeta inversed ($1/\zeta$) is equal to $(2\pi mkT/h^2)^{3/2}V/N$) or n_t^3 is the thermodynamic potential (Gibbs function or Gibbs energy) and that n_t^3 is thus obtained as a function of ($T^{3/2}V/N$), one also identified mentioned above by Boltzmann for defining entropy and that the partial derivative of $kT\ln Z$ (Schrödinger's ψ function) with respect to $1/n_t^3$.

Schrödinger (1952) further remarked that at high temperature and low density, conditions similar to those given here for the Carnot cycle, that "the quantum cells are so numerous, that on average, even in the most populated region only every 10,000th or 100,000th is occupied at all, … the occupancy is either zero (most of them) or 1, hardly ever 2". He computes for monatomic helium at 0 °C and 1 atm that $1/\zeta$ (= n_t^3) is equal to 255,570. "For translation, even at 2-3 K and 100th the volume, this factor $1/\zeta$ (n_t^3) is still about 100,000".

Then for an ideal monatomic gas, from equation (vii)

$$S = N\varepsilon_j/T + kN\ln z \; = \; E/T + \; k\ln z^N$$

We have

$$S = E + k\ln Z = (E - A)/T \qquad \text{(xvii)}$$

A $= G - RT$ (when N is one mole)

$= E - ST$

G $= H - ST$

For diatomic molecules like N_2, we have the microcanonical partition function including a rotational term.

$$z = z_t z_r = \mathrm{N} n_t^3 j_r^2 \qquad \text{(xviii)}$$

From the same relationship as equation (xvi) we have equation (xix).

$$Z = (n_t^3 j_r^2 e\mathrm{N})^{\mathrm{N}}/\mathrm{N}^{\mathrm{N}} = (n_t^3 e\, j_r^2)^{\mathrm{N}}$$

$$G = H - ST$$

These relationships regarding translational entropy for monatomic systems apply for translation to all polyatomic gases; they are particularly important as the Gibbs energy *G* for determining chemical potential or distance from equilibrium. For polyatomic gases, the partition functions as action for rotation and vibration are easily added (Kennedy et al., 2019), allowing determination of the total entropy.

For molecules like N_2, vibrational entropy is negligible even at 640K. However, in the case of CO_2 for the vibration resonant with quanta at 15 µm wavelength of importance in greenhouse forcing, some 22% of the molecules are excited to the first level of $3hv/2$, making a significant contribution to the entropy (Table). As shown in the table, the excited vibrational levels can also be expressed in terms of translational action and entropy using the formula *mrv* for the rarer excited species. Note the larger radii r_n of activated species, indicating their low probability but greater impulse δmrv in collisions.

Table. Vibrational energy (ε) states for N_2 and CO_2 indicating translational action of molecules excited up to $\varepsilon_{vib} = 5hv/2$.

**640 K N_2**	**N_0/N_n $=V_n/V_o$**	**δε ergs**	**r_n/r_o= $@_{tn}/@_{t0}$**	**ε ex action $kT\ln(n_{tn}/n_{to})^3$**	**288 K N_2**	**N_0/N_n $=V_n/V_o$**	**r_n/r_o= $@_{tn}/@_{t0}$**	**ε ex action $kT\ln(n_{tn}/n_{to})^3$**
$\delta\varepsilon_{vib}$		$\times10^{13}$		$\times10^{13}$	$\delta\varepsilon_{vib}$		$\times10^{13}$	$\times10^{13}$
$e^{-2hv/kT}$	102582	10.1954	46.812	10.1954	$5hv/2$	1.367e[11]	5151.40	10.1954
$e^{-hv/kT}$	320.284	5.0977	6.842	5.0977	$3hv/2$	369605	77.771	5.0977
e^{o}	1.000	0	1.000	0	$hv/2$	0	1.000	0

640 K CO_2	N_o/N_n $=V_n/V_o$	δε ergs	r_n/r_o= $@_{tn}/@_{t0}$	ε ex action $kT\ln(n_{tn}/n_{to})^3$	288 K CO_2	N_o/N_n $=V_n/V_o$	r_n/r_o= $@_{tn}/@_{t0}$	ε ex action $kT\ln(n_{tn}/n_{to})^3$
$\delta\varepsilon_{vib}$					$\delta\varepsilon_{vib}$		$\times10^{13}$	$\times10^{13}$
$e^{-2hv/kT}$	20.065	2.6499	2.717	2.6499	$e^{-2hv/kT}$	783.99	9.221	2.6499
$e^{-hv/kT}$	4.479	1.3250	1.648	1.3250	$e^{-hv/kT}$	28.000	3.037	1.3250
e^{o}	1.000	0	1.000	0	e^{o}	1.000	1.000	0
