# Supplementary material for: Action and Entropy in Heat Engines: An Action Revision of the Carnot Cycle
Source: Entropy (Basel). 2021 Jul 5;23(7):860. doi: 10.3390/e23070860 (PMC8304742; doi:10.3390/e23070860)
Supplement: Supplementary file 1 [file entropy-23-00860-s001.zip › supplementary-2.pdf]

```
CARNOT6   Carnot Cycle Ar,N2,H2O
Corrected 04 Apr 2021
Inputs Y,F,R,P,H,L,M,N,A,B,C,Q
Input rotational symmetry
0160     096     LABEL
0161     089     Y
0162     107     STO MEM
0163     056       8
0164     057       9
0165     119     print
0166     002     HALT
0167     048       0
Source temperature degrees Kelvin Ts
0208     096     LABEL
0209     070     F
0210     107     STO MEM
0211     048       0
0212     049       1
0213     119     print
0214     002     HALT
0215     048       0
Temperature of refrigerator Tr
0256     096     LABEL
0257     082     R
0258     107     STO MEM
0259     048       0
0260     050       2
0261     119     print
0262     002     HALT
0263     048       0
Pressure in atm
0304     096     LABEL
0305     080     P
0306     107     STO MEM
0307     048       0
0308     051       3
0309     119     print
0310     002     HALT
0311     048       0
Stow  heat capacity Cv/R
0352     096     LABEL
0353     072     H
0354     107     STO MEM
0355     048       0
0356     052       4
0357     119     print
0358     002     HALT
0359     048       0
Atom mass H1 polyatomic
0400     096     LABEL
0401     076     L
0402     107     STO MEM
0403     048       0
0404     055       7
0405     119     print
0406     002     HALT
Atom monatomic Ar, or M1 or central M
0447     096     LABEL
0448     077     M
0449     107     STO MEM
0450     048       0
0451     053       5
0452     119     print
0453     002     HALT
0454     048       0
Stow molecular mass 2 N or O
0495     096     LABEL
0496     078     N
0497     107     STO MEM
0498     049       1
0499     053       5
0500     119     print
0501     002     HALT
0502     048       0
Bond length diatomic
0543     096     LABEL
0544     090     Z
0545     107     STO MEM
0546     049       1
0547     054       6
0548     119     print
0549     002     HALT
0550     048       0
Moment 1 polyatomic
0591     096     LABEL
0592     065     A
0593     107     STO MEM
0594     048       0
0595     056       8
0596     119     print
0597     002     HALT
Moment 2 polyatomic
0638     096     LABEL
0639     066     B
0640     107     STO MEM
0641     048       0
0642     057       9
0643     119     print
0644     002     HALT
Stow moment 3 polyatomic
0685     096     LABEL
0686     067     C
0687     107     STO MEM
0688     049       1
0689     048       0
0690     119     print
0691     002     HALT
Input source heat nkT=Qf ergs/molecule
Input n for nkT1
0772     096     LABEL
0773     081     Q
0774     107     STO MEM
0775     048       0
0776     054       6
0777     119     print
0778     002     HALT
Execute <SH>X
0819     096     LABEL
0820     088     X
0821     049       1
```

```
0822    048     0
0823    046     .
0824    050     2
0825    050     2
0826    057     9
0827    054     6
0828    055     7
0829    050     2
0830    119     print
Stow zt translational correction
0871    107     STO MEM
0872    057      9
0873    057      9
Input Boltzmann's k
0914    049     1
0915    046     .
0916    051     3
0917    056     8
0918    048     0
0919    054     6
0920    050     2
0921    053     5
0922    057     9
0923    101     EE
0924    049     1
0925    054     6
0926    111     +/-
0927    107     STO MEM
0928    057      9
0929    055      7
0930    119     print
0931    049      1
0932    046      .
0933    048     0
0934    053     5
0935    052     4
0936    053     5
0937    057     9
0938    049     1
0939    056     8
0940    053     5
0941    101     EE
0942    050     2
0943    055     7
0944    111     +/-
0945    107     STO MEM
0946    057      9
0947    056      8
0948    119     print
Input mass H atom
0989    049      1
0990    046      .
0991    054     6
0992    055     7
0993    050     2
0994    054     6
0995    049     1
0996    052     4
0997    101     EE
0998    050     2
0999    052      4
1000    111     +/-
1001    107     STO MEM
1002    057      9
1003    054      6
1004    119     print
Input c speed light
1045    050      2
1046    046      .
1047    057      9
1048    057      9
1049    055      7
1050    057      9
1051    050      2
1052    052      4
1053    053      5
1054    056      8
1055    101      EE
1056    049      1
1057    048      0
1058    107     STO MEM
1059    057      9
1060    053      5
Recall pressure1 and calculate r1
r1^3=(T1p2r2^3)/T2p1
1141    040      (
1142    109     RCL MEM
1143    048      0
1144    049      1
1145    047      /
1146    050      2
1147    057      9
1148    056      8
1149    046      .
1150    049      1
1151    053      5
1152    041      )
1153    047      /
1154    109     RCL MEM
1155    048      0
1156    051      3
1157    061      =
1158    092     Root
1159    051      3
1160    042      x
Input r2 at 1 atm and 298.15K
1201    049      1
1202    046      .
1203    054      6
1204    057      9
1205    057      9
1206    054      6
1207    052      4
1208    101      EE
1209    055      7
1210    111      +/-
1211    041      )
1212    119     print
Store r1 radial separation
1253    107     STO MEM
```

```
1254    057     9
1255    048     0
Go to S
1296    083    S
Subroutine SnTn entropic energy
1337    096    LABEL
1338    068    D
1339    040     (
1340    040     (
1341    109    RCL MEM
1342    048     0
1343    053     5
1344    043     +
1345    109    RCL MEM
1346    049     1
1347    053     5
1348    041     )
1349    042     x
1350    109    RCL MEM
1351    057     9
1352    054     6
1353    042     x
1354    040     (
1355    109    RCL MEM
1356    053     5
1357    048     0
1358    113    Square
1359    041     )
1360    061     =
Print In
1401    119    print
1402    042     x
1403    051     3
1404    042     x
1405    109    RCL MEM
1406    057     9
1407    055     7
1408    042     x
Recall temperature r51
1449    109    RCL MEM
1450    053     5
1451    049     1
1452    041     )
1453    047     /
1454    109    RCL MEM
1455    057     9
1456    057     9
1457    061     =
Print 3kTnIn=@tn^2 trans action square
1498    119    print
1499    092    Root
1500    050     2
1501    061     =
Print @n translational action
1542    119    print
1543    047     /
1544    109    RCL MEM
1545    057     9
1546    056     8
1547    061     =
Print quantum number
1588    119    print
1589    107    STO MEM
1590    054     6
1591    048     0
1592    121    Cube
1593    110    LOG e
1594    042     x
1595    109    RCL MEM
1596    057     9
1597    055     7
1598    061     =
Print translational -gt/T
1639    119    print
1640    107    STO MEM
1641    055     7
1642    048     0
1643    042     x
1644    109    RCL MEM
1645    053     5
1646    049     1
Print kTnln[@nt/h-)^3/zt]=-gtn
1687    061     =
1688    119    print
1689    107    STO MEM
1690    055     7
1691    049     1
1692    043     +
1693    040     (
1694    109    RCL MEM
1695    057     9
1696    055     7
1697    042     x
1698    109    RCL MEM
1699    053     5
1700    049     1
1701    041     )
1702    061     =
Print -a Helmholtz energy
1743    119    print
1744    107    STO MEM
1745    055     7
1746    054     6
1747    043     +
1748    040     (
1749    109    RCL MEM
1750    048     0
1751    052     4
1752    042     x
1753    109    RCL MEM
1754    057     9
1755    055     7
1756    042     x
1757    109    RCL MEM
1758    053     5
1759    049     1
1760    041     )
1761    061     =
1762    119    print
Print stnTn for any gas
```

```
1803      107      STO MEM
1804      056        8
1805      048        0
1806      047        /
1807      109      RCL MEM
1808      053        5
1809      049        1
1810      061        =
Print stn
1851      119      print
1852      107      STO MEM
1853      056        8
1854      049        1
1855      040        (
1856      109      RCL MEM
1857      055        7
1858      049        1
1859      047        /
1860      109      RCL MEM
1861      054        6
1862      048        0
1863      061        =
Print virtual hv
1904      119      print
1905      047        /
1906      109      RCL MEM
1907      057        9
1908      056        8
1909      047        /
1910      050        2
1911      047        /
1912      112        pi
1913      061        =
Print virtual translational freq v
1954      119      print
1955      114        1/x
1956      042        x
1957      109      RCL MEM
1958      057        9
1959      053        5
1960      061        =
1961      119      print
Print wavelength virtual trans. quanta
Test if diatomic
2042      109      RCL MEM
2043      049        1
2044      053        5
2045      038      If zero
2046      084      T
Test if triatomic H2O
2087      109      RCL MEM
2088      048        0
2089      056        8
2090      038      If zero
2091      069      E
Test if non-linear
2132      040        (
2133      048        0
2134      045        -
2135      109      RCL MEM
```

```
2136      049        1
2137      050        2
2138      041        )
2139      064      Inverse
2140      037      If pos
2141      116      Tangent
Calculate Sr for water
Sr=Rln[pi^/2e^3/2@a@b@c/h-^3/z)
Calculate @rA
2262      096      LABEL
2263      116      Tangent
2264      040        (
2265      109      RCL MEM
2266      048        0
2267      056        8
2268      042        x
2269      109      RCL MEM
2270      053        5
2271      049        1
2272      042        x
2273      050        2
2274      042        x
2275      109      RCL MEM
2276      057        9
2277      055        7
2278      041        )
2279      064      Inverse
2280      113      Square
2281      061        =
Print, stow @rA in r17
2322      119      print
2323      107      STO MEM
2324      049        1
2325      055        7
2326      109      RCL MEM
2327      049        1
2328      055        7
2329      047        /
Recall h-
2370      109      RCL MEM
2371      057        9
2372      056        8
2373      061        =
Print, stow @rA/h- in r17
2414      119      print
2415      107      STO MEM
2416      049        1
2417      055        7
Calculate @rB
2458      109      RCL MEM
2459      048        0
2460      057        9
2461      042        x
2462      109      RCL MEM
2463      053        5
2464      049        1
2465      042        x
2466      050        2
Recall k
2507      042        x
```

```
2508    109    RCL MEM
2509    057      9
2510    055      7
2511    041      )
2512    092    Root
2513    050      2
2514    061      =
Print, stow ∂rB in r18
2555    119    print
2556    107    STO MEM
2557    049      1
2558    056      8
2559    109    RCL MEM
2560    049      1
2561    056      8
2562    047      /
Recall h-
2603    109    RCL MEM
2604    057      9
2605    056      8
2606    061      =
Print, stow ∂rB/h- in r18
2647    119    print
2648    107    STO MEM
2649    049      1
2650    056      8
2651    061      =
Calculate ∂rC
2692    040      (
2693    109    RCL MEM
2694    049      1
2695    048      0
2696    042      x
2697    109    RCL MEM
2698    053      5
2699    049      1
2700    042      x
2701    050      2
2702    042      x
Recall k
2743    109    RCL MEM
2744    057      9
2745    055      7
2746    041      )
2747    092    Root
2748    050      2
2749    061      =
Print. stow ∂rC in r19
2790    119    print
2791    107    STO MEM
2792    049      1
2793    057      9
2794    109    RCL MEM
2795    049      1
2796    057      9
2797    047      /
Recall h-
2838    109    RCL MEM
2839    057      9
2840    056      8
2841    061      =
Print, stow ∂rC/h-
2882    119    print
2883    107    STO MEM
2884    049      1
2885    057      9
jrxjrxjr
2926    040      (
2927    040      (
2928    109    RCL MEM
2929    049      1
2930    055      7
2931    042      x
2932    109    RCL MEM
2933    049      1
2934    056      8
2935    042      x
2936    109    RCL MEM
2937    049      1
2938    057      9
2939    041      )
Print jrA x jrB x jrC
2980    119    print
Recall symmetry factor
3021    042      x
3022    040      (
3023    112      pi
3024    064    Inverse
3025    113    Square
3026    041      )
3027    041      )
Print corrected jrcubed
3068    119    print
3069    107    STO MEM
3070    054      6
3071    049      1
3072    109    RCL MEM
3073    054      6
3074    049      1
3075    047      /
3076    109    RCL MEM
3077    056      8
3078    057      9
3079    110    LOG e
3080    042      x
Recall k
3121    109    RCL MEM
3122    057      9
3123    055      7
3124    061      =
Print, stow -gr/T in r72
3165    119    print
3166    107    STO MEM
3167    055      7
3168    050      2
3169    119    print
3170    042      x
3171    109    RCL MEM
3172    053      5
3173    049      1
```

```
3174    061         =
Print -gr
3215    119    print
3216    107    STO MEM
3217    055      7
3218    051      3
3219    040      (
3220    109    RCL MEM
3221    055      7
3222    049      1
3223    043      +
3224    109    RCL MEM
3225    055      7
3226    051      3
3227    041      )
3228    061      =
Print -gt -gr
3269    119    print
3270    107    STO MEM
3271    055      7
3272    052      4
3273    047      /
3274    109    RCL MEM
3275    053      5
3276    049      1
3277    061      =
Print -grn/Tn - gtn/Tn
3318    119    print
3319    107    STO MEM
3320    055      7
3321    053      5
3322    040      (
3323    109    RCL MEM
3324    056      8
3325    048      0
3326    043      +
3327    109    RCL MEM
3328    055      7
3329    051      3
3330    041      )
Print total snTn
3371    119    print
3372    107    STO MEM
3373    056      8
3374    050      2
3375    047      /
3376    109    RCL MEM
3377    053      5
3378    049      1
3379    061      =
Print stn + Srn
3420    119    print
3421    040      (
3422    109    RCL MEM
3423    055      7
3424    051      3
3425    047      /
3426    109    RCL MEM
3427    054      6
3428    049      1
3429    041      )
3430    061      =
Print virtual hvr
3471    119    print
3472    047      /
3473    109    RCL MEM
3474    057      9
3475    056      8
3476    047      /
3477    050      2
3478    047      /
3479    112      pi
3480    061      =
Print vr
3521    119    print
3522    114      1/x
3523    042      x
3524    109    RCL MEM
3525    057      9
3526    053      5
3527    061      =
Print virtual rotational wavelength
3568    119    print
3569    063    Goto
3570    084    T
Calculate rotational quantum no.
3611    096    LABEL
3612    069    E
3613    109    RCL MEM
3614    048      0
3615    053      5
3616    042      x
3617    109    RCL MEM
3618    049      1
3619    053      5
3620    041      )
3621    047      /
3622    040      (
3623    109    RCL MEM
3624    048      0
3625    053      5
3626    043      +
3627    109    RCL MEM
3628    049      1
3629    053      5
3630    041      )
3631    042      x
Recall H atom mass
3672    109    RCL MEM
3673    057      9
3674    054      6
3675    042      x
3676    040      (
3677    109    RCL MEM
3678    049      1
3679    054      6
3680    113    Square
3681    041      )
3682    061      =
Print Ir
```

```
3723     119     print
3724     042       x
3725     040       (
3726     050       2
3727     042       x
3728     109     RCL MEM
3729     057       9
3730     055       7
3731     042       x
3732     109     RCL MEM
3733     053       5
3734     049       1
3735     041       )
3736     061       =
3737     047       /
3738     109     RCL MEM
3739     056       8
3740     057       9
3741     061       =
Print ∂r^2
3782     119     print
3783     064     Inverse
3784     113     Square
3785     061       =
Print ∂r rotational action
3826     119     print
3827     047       /
3828     109     RCL MEM
3829     057       9
3830     056       8
3831     061       =
Print jr
3872     119     print
3873     107     STO MEM
3874     054       6
3875     049       1
3876     109     RCL MEM
3877     054       6
3878     049       1
3879     110     LOG e
3880     042       x
3881     109     RCL MEM
3882     057       9
3883     055       7
3884     042       x
3885     050       2
3886     061       =
Print rotational -gr/T
3927     107     STO MEM
3928     055       7
3929     050       2
3930     119     print
3931     042       x
3932     109     RCL MEM
3933     053       5
3934     049       1
3935     061       =
Print -gr
3976     119     print
3977     107     STO MEM
3978     055       7
3979     051       3
3980     040       (
3981     109     RCL MEM
3982     055       7
3983     049       1
3984     043       +
3985     109     RCL MEM
3986     055       7
3987     051       3
3988     041       )
3989     061       =
Print total -gt - gr
4030     119     print
4031     107     STO MEM
4032     055       7
4033     052       4
4034     047       /
4035     109     RCL MEM
4036     053       5
4037     049       1
4038     061       =
Print -grn/Tn - gtn/Tn
4079     119     print
4080     107     STO MEM
4081     055       7
4082     053       5
4083     040       (
4084     109     RCL MEM
4085     056       8
4086     048       0
4087     043       +
4088     109     RCL MEM
4089     055       7
4090     051       3
4091     041       )
Print Total snTn
4132     107     STO MEM
4133     056       8
4134     050       2
4135     119     print
4136     047       /
4137     109     RCL MEM
4138     053       5
4139     049       1
4140     061       =
Print stn+srn
4181     119     print
4182     040       (
4183     109     RCL MEM
4184     055       7
4185     051       3
4186     047       /
4187     109     RCL MEM
4188     054       6
4189     049       1
4190     041       )
4191     061       =
Print virtual hvr
4232     119     print
```

```
4233     047      /
4234     109     RCL MEM
4235     057      9
4236     056      8
4237     047      /
4238     050      2
4239     047      /
4240     112      pi
4241     061      =
Print vr
4282     119     print
4283     114      1/x
4284     042      x
4285     109     RCL MEM
4286     057      9
4287     053      5
4288     061      =
Print virtual rotational wavelength
4329     119     print
4330     096     LABEL
4331     084     T
4332     109     RCL MEM
4333     053      5
4334     048      0
4335     121     Cube
4336     042      x
4337     056      8
4338     061      =
Print vn
4379     119     print
4380     107     STO MEM
4381     054      6
4382     048      0
4383     040      (
4384     109     RCL MEM
4385     055      7
4386     049      1
4387     047      /
4388     109     RCL MEM
4389     054      6
4390     048      0
4391     041      )
Print -gt/a^3 density Gibbs
4432     119     print
4433     040      (
4434     109     RCL MEM
4435     055      7
4436     052      4
4437     047      /
4438     109     RCL MEM
4439     054      6
4440     048      0
4441     041      )
Print -gt+-gr/a^3 Gibbs density
4482     119     print
4483     040      (
4484     109     RCL MEM
4485     055      7
4486     054      6
4487     047      /
4488     109     RCL MEM
4489     054      6
4490     048      0
4491     041      )
Print -at/a^3 Helmholtz density
4532     119     print
4533     040      (
4534     109     RCL MEM
4535     057      9
4536     055      7
4537     042      x
4538     109     RCL MEM
4539     053      5
4540     049      1
4541     041      )
4542     047      /
4543     040      (
4544     109     RCL MEM
4545     053      5
4546     048      0
4547     121     Cube
4548     041      )
4549     047      /
4550     056      8
4551     061      =
Print pressure pn
4592     119     print
4593     042      x
4594     109     RCL MEM
4595     054      6
4596     048      0
4597     061      =
Print pnvn
4638     119     print
4639     047      /
4640     109     RCL MEM
4641     053      5
4642     049      1
4643     061      =
Print pnvn/Tn
4684     119     print
4685     060     Return
Set up V values
4726     096     LABEL
4727     083     S
Calculate Q1 source heat per molecule
4768     040      (
Input n for Qs
4809     109     RCL MEM
4810     048      0
4811     054      6
4812     042      x
4813     109     RCL MEM
4814     057      9
4815     055      7
4816     042      x
4817     109     RCL MEM
4818     048      0
4819     049      1
4820     041      )
```

```
Print Qf
4861     119     print
4862     047       /
4863     109     RCL MEM
4864     048       0
4865     049       1
4866     061       =
Print ds/molecule=kln(V2/V1)
4907     119     print
4908     047       /
4909     109     RCL MEM
4910     057       9
4911     055       7
4912     061       =
4913     064     Inverse
4914     110     LOG e
4915     061       =
Print V2/V1 = V3/V4
4956     119     print
4957     107     STO MEM
4958     053       5
4959     051       3
Calculate V2/V3=(Tr/Ts)^Cv/nR
5000     040       (
5001     109     RCL MEM
5002     048       0
5003     050       2
5004     047       /
5005     109     RCL MEM
5006     048       0
5007     049       1
5008     041       )
5009     091     Exponent
5010     109     RCL MEM
5011     048       0
5012     052       4
5013     061       =
Print V2/V3
5054     119     print
5055     114      1/x
5056     042       x
5057     109     RCL MEM
5058     053       5
5059     051       3
5060     061       =
Print V3/V1
5101     119     print
5102     107     STO MEM
5103     053       5
5104     052       4
Calculate S1T1
5145     109     RCL MEM
5146     048       0
5147     049       1
5148     107     STO MEM
5149     053       5
5150     049       1
5151     109     RCL MEM
5152     057       9
5153     048       0
5154     107     STO MEM
5155     053       5
5156     048       0
Go to Label D and return
5197     068     D
5198     109     RCL MEM
5199     053       5
5200     051       3
5201     064     Inverse
5202     121     Cube
5203     042       x
5204     109     RCL MEM
5205     057       9
5206     048       0
5207     061       =
Print r2
5248     119     print
5249     107     STO MEM
5250     053       5
5251     048       0
5252     068     D
5253     109     RCL MEM
5254     053       5
5255     052       4
5256     064     Inverse
5257     121     Cube
5258     042       x
5259     109     RCL MEM
5260     057       9
5261     048       0
5262     061       =
Print r3
5303     119     print
5304     107     STO MEM
5305     053       5
5306     048       0
5307     109     RCL MEM
5308     048       0
5309     050       2
5310     107     STO MEM
5311     053       5
5312     049       1
5313     068     D
5314     109     RCL MEM
5315     053       5
5316     048       0
5317     121     Cube
5318     042       x
5319     040       (
5320     109     RCL MEM
5321     053       5
5322     051       3
5323     114      1/x
5324     041       )
5325     061       =
5326     064     Inverse
5327     121     Cube
5328     061       =
Print r4
5369     119     print
```
